# Individual participant data meta‐analysis to examine interactions between treatment effect and participant‐level covariates: Statistical recommendations for conduct and planning

**DOI:** 10.1002/sim.8516

**Published:** 2020-04-30

**Authors:** Richard D. Riley, Thomas P.A. Debray, David Fisher, Miriam Hattle, Nadine Marlin, Jeroen Hoogland, Francois Gueyffier, Jan A. Staessen, Jiguang Wang, Karel G.M. Moons, Johannes B. Reitsma, Joie Ensor

**Affiliations:** ^1^ Centre for Prognosis Research, School of Primary, Community and Social Care Keele University Staffordshire UK; ^2^ Julius Center for Health Sciences and Primary Care University Medical Center Utrecht Utrecht The Netherlands; ^3^ MRC Clinical Trials Unit, Institute of Clinical Trials & Methodology, Faculty of Population Health Sciences University College London London UK; ^4^ Blizard Institute, Barts and The London School of Medicine and Dentistry Queen Mary University of London London UK; ^5^ Inserm Lyon France; ^6^ Department of Cardiovascular Sciences, Research Unit Hypertension and Cardiovascular Epidemiology, Studies Coordinating Centre KU Leuven Leuven Belgium; ^7^ Centre for Epidemiological Studies and Clinical Trials, Ruijin Hospital Shanghai Jiaotong University School of Medicine Shanghai China

**Keywords:** effect modifier, individual participant data (IPD), meta‐analysis, subgroup effect, treatment‐covariate interaction

## Abstract

Precision medicine research often searches for treatment‐covariate interactions, which refers to when a treatment effect (eg, measured as a mean difference, odds ratio, hazard ratio) changes across values of a participant‐level covariate (eg, age, gender, biomarker). Single trials do not usually have sufficient power to detect genuine treatment‐covariate interactions, which motivate the sharing of individual participant data (IPD) from multiple trials for meta‐analysis. Here, we provide statistical recommendations for conducting and planning an IPD meta‐analysis of randomized trials to examine treatment‐covariate interactions. For conduct, two‐stage and one‐stage statistical models are described, and we recommend: (i) interactions should be estimated directly, and not by calculating differences in meta‐analysis results for subgroups; (ii) interaction estimates should be based solely on within‐study information; (iii) continuous covariates and outcomes should be analyzed on their continuous scale; (iv) nonlinear relationships should be examined for continuous covariates, using a multivariate meta‐analysis of the trend (eg, using restricted cubic spline functions); and (v) translation of interactions into clinical practice is nontrivial, requiring individualized treatment effect prediction. For planning, we describe first why the decision to initiate an IPD meta‐analysis project should not be based on between‐study heterogeneity in the overall treatment effect; and second, how to calculate the power of a potential IPD meta‐analysis project in advance of IPD collection, conditional on characteristics (eg, number of participants, standard deviation of covariates) of the trials (potentially) promising their IPD. Real IPD meta‐analysis projects are used for illustration throughout.

## 1 INTRODUCTION

Precision medicine and stratified healthcare tailors treatment decisions to individuals based on their particular characteristics.^1^, ^2^ The goal is to optimize treatment decisions and reduce unnecessary costs for each individual, by selecting treatments most likely to benefit (or least likely to harm) them based on their participant‐level covariate values. For example, trastuzumab is only given to the subgroup of breast cancer patients who are human epidermal growth factor receptor 2 (HER‐2) positive, as it is known to lock on to the HER‐2 protein, block the receptor, and stop the cells from dividing and growing.^3^ It is therefore considered unnecessary for those who are HER‐2 negative. In this situation, there is a so‐called treatment‐covariate interaction, such that the treatment effect (measured on a scale such as a mean difference, risk ratio, odds ratio, or hazard ratio) changes according to an individual's HER‐2 status. Participant‐level covariates that interact with treatment effect are also known as effect modifiers, moderators, predictors of treatment effect and, mainly in the cancer literature, predictive markers.

Though some treatment‐covariate interactions, such as HER‐2, are suspected in advance due to strong biological rationale, others are only identified following secondary investigations of existing data. Single randomized trials are typically powered on the overall treatment effect (ie, the treatment effect averaged across all individuals), and so do not usually have sufficient power to detect differences in treatment effect between individuals. Powering a single trial to detect a genuine treatment‐covariate interaction will typically require at least four times the sample size needed to test the overall treatment effect;^4^ thus the funding of such trials is expensive and often infeasible. When individual participant data (IPD) from multiple randomized trials are available, meta‐analysis provides the opportunity to increase power to detect true treatment‐covariate interactions.^5^ For this reason, many IPD meta‐analyses of randomized trials are initiated specifically to examine one or more potential treatment‐covariate interactions, often with the aim to reduce or explain observed between‐study heterogeneity in a previous meta‐analysis of (published) aggregate data. For pharmaceutical companies, a driver may be to rescue a treatment that previously showed no overall benefit on all patients combined, but which may still benefit a select group of individuals.

Over the last decade we have been involved in a number of IPD meta‐analysis projects aiming to examine treatment‐covariate interactions at the participant‐level, and learnt important lessons and pitfalls from a statistical point of view. In this tutorial article, we share our experience to help other researchers in this situation, building on previous work in both IPD meta‐analysis and single study settings.^4^, ^6^, ^7^, ^8^, ^9^, ^10^, ^11^, ^12^, ^13^, ^14^, ^15^, ^16^ Our main goal is to provide statistical recommendations for the meta‐analysis part of the project; in particular, how to estimate treatment‐covariate interactions using either a one‐stage or two‐stage IPD meta‐analysis framework. A further aim is to highlight two statistical topics that are relevant to consider before embarking on an IPD meta‐analysis project to examine treatment‐covariate interactions. The outline of the article is as follows. Section 2 describes two motivating examples used for illustration throughout the whole article. Section 3 describes a framework for two‐stage and one‐stage IPD meta‐analysis models for estimating treatment‐covariate interactions, and Section 4 highlights five recommendations in this context: (i) calculating differences in meta‐analysis results for subgroups is misleading; (ii) interaction estimates should be based solely on within‐study information; (iii) continuous covariates (and outcomes) should be analyzed on their continuous scale; (iv) nonlinear relationships should be examined for continuous covariates, using a multivariate meta‐analysis of the trend (eg, using restricted cubic spline functions); and (v) translation of interactions into clinical practice requires individualized treatment effect prediction. Section 5 then describes two statistical issues in guiding decisions to initiate an IPD meta‐analysis project to examine interactions. First, how the decision to initiate such IPD meta‐analysis projects should not be based on the amount of between‐study heterogeneity in the overall treatment effect. Second, how to calculate, in advance of IPD collection, the power of a potential IPD meta‐analysis conditional on the characteristics of those trials promising their IPD. Section 6 concludes with discussion.

## 2 MOTIVATING EXAMPLES

We now introduce two examples, which will be used to illustrate some of the key issues in later sections.

### 2.1 An IPD meta‐analysis examining the effect of antihypertensive treatment

Wang et al^17^ performed an IPD meta‐analysis of trials in participants with hypertension to investigate to what extent lowering of systolic blood pressure (SBP) contributed to the prevention of cardiovascular events. They selected randomized trials that tested active antihypertensive drugs against control. IPD was sought from trials in the INdividual Data ANalysis of Antihypertensive intervention trials (INDANA) dataset or at the Studies Coordinating Centre in Leuven (Belgium). Ten trials (with a parallel group design) were ultimately included, and these provided IPD for a total of 28 581 patients. Key outcomes of interest by end of follow‐up were SBP, cardiovascular disease (CVD), and all‐cause mortality. An important focus is on whether the effect of antihypertensive treatment on these outcomes is modified by (ie, interacts with) participant‐level covariates such as gender and baseline blood pressure.

### 2.2 An IPD meta‐analysis examining the effect of interventions to reduce gestational weight gain in pregnancy

The International Weight Management in Pregnancy (i‐WIP) Collaborative Group obtained IPD from 36 trials (12 447 women), to investigate whether diet and lifestyle interventions improve outcomes during pregnancy. A primary outcome was whether interventions reduced gestational weight gain, for which 33 studies and a total of 9320 women were available. Weight was recorded as a continuous outcome (in kg), available at baseline (ie, confirmation of pregnancy) and follow‐up (ie, last available weight recorded before delivery). Other outcomes include pre‐eclampsia and stillbirth. A key aim was to examine if the intervention effect on these maternal and fetal outcomes is modified by (ie, interacts with) participant‐level covariates such as the mother's age and body mass index (BMI) at baseline.

## 3 TWO‐STAGE AND ONE‐STAGE IPD META‐ANALYSIS MODELS FOR ESTIMATING TREATMENT‐COVARIATE INTERACTIONS

In this section, we introduce the framework of two‐stage and one‐stage models for conducting an IPD meta‐analysis to examine treatment‐covariate interactions based on within‐study information.

### 3.1 The two‐stage approach

A two‐stage IPD meta‐analysis for summarizing treatment‐covariate interactions is a straightforward application of a traditional meta‐analysis framework. In the first stage the treatment‐covariate interactions are estimated using the IPD from each trial separately; then in the second stage these interaction estimates are pooled using a traditional (eg, inverse‐variance weighted) meta‐analysis model.

Consider an IPD meta‐analysis of multiple randomized trials, each comparing the effect of a particular treatment relative to a control using a simple parallel group design. Let *i* denote trial (*i* = 1 to *S*), *n*_*i*_ denote the number of participants in the *i*th trial, *j* denote participant (*j* = 1 to *n*_*i*_), and *x*_*ij*_ denote allocation to either the treatment (*x*_*ij*_ = 1) or control (*x*_*ij*_ = 0) group for the *j*th participant in the *i*th trial. Let *z*_*ij*_ be a key participant‐level covariate of interest (eg, the sex of participant *j* in trial *i*), observed for all participants in each trial. Then, the two‐stage approach can be detailed as follows.

#### 3.1.1 First stage

The analysis to apply in the first stage depends on the outcome type. For example, assume the aim is to evaluate the treatment effect on a continuous outcome (*y*_*ij*_), such as SBP, then the first stage of the IPD meta‐analysis should apply a linear regression *in each trial separately*, adjusting for the baseline SBP (*y*_0*ij*_) and the covariate (*z*_*ij*_), whilst including the treatment (*x*_*ij*_) and the treatment‐covariate interaction (*x*_*ij*_*z*_*ij*_):

(1) $$\begin{array}{cc} y_{\mathrm{ij}}=\; & \alpha_{i}+\beta_{1i}z_{\mathrm{ij}}+\beta_{2i}x_{\mathrm{ij}}+\beta_{3i}y_{0\mathrm{ij}}+\gamma_{W\;i}x_{\mathrm{ij}}z_{\mathrm{ij}}+e_{\mathrm{ij}}\\ & e_{\mathrm{ij}}∼N\left(0,,,\sigma_{i}^{2}\right). \end{array}$$

The key parameter in model (1) is the treatment‐covariate interaction (*γ*_*Wi*_), which represents the change in the treatment effect (ie, the change in the mean difference in outcome value for treatment compared to control) for a 1‐unit increase in *z*_*ij*_ after adjusting for the prognostic effects of *z*_*ij*_ and *y*_0*ij*_. Other terms are *α*_*i*_ (the intercept, that is, the expected outcome value for participants in the control group with zero values of *z*_*ij*_ and *y*_0*ij*_), *β*_1*i*_ and *β*_3*i*_ (the expected change in the outcome value for a 1‐unit increase in *z*_*ij*_ and *y*_0*ij*_, respectively) and *β*_2*i*_ (the treatment effect for those with a *z*_*ij*_ of zero after adjusting for baseline). The same residual variance ($\sigma_{i}^{2}$) is assumed for the treatment and control groups, but this can be relaxed if considered appropriate.^18^

If a binary outcome was rather of interest (ie, *y*_*ij*_ = 0 or 1), then alternatively a binomial regression could be fitted in each trial separately, such as a logistic regression,

(2) $$\begin{array}{cc} & y_{\mathrm{ij}}∼\text{Bernoulli}\left(p_{\mathrm{ij}}\right)\\ & \ln\left(\frac{p_{\mathrm{ij}}}{1-p_{\mathrm{ij}}}\right)=\alpha_{i}+\beta_{1i}z_{\mathrm{ij}}+\beta_{2i}x_{\mathrm{ij}}+\beta_{3i}y_{0\mathrm{ij}}+\gamma_{W\;i}x_{\mathrm{ij}}z_{\mathrm{ij}}, \end{array}$$

where *p*_*ij*_ is the probability of an outcome event (ie, *y*_*ij*_ = 1) for the *j*th participant in the *i*th trial conditional on their covariate values. The parameters are defined similar to those for model (1), expect now we model the log‐odds of the outcome event (ie, $\ln\left(\frac{p_{\mathrm{ij}}}{1-p_{\mathrm{ij}}}\right)$) such that the treatment effect is measured by a log odds ratio. Hence, the treatment‐covariate interaction (*γ*_*Wi*_) represents the change in the log odds ratio for a 1‐unit increase in *z*_*ij*_ after adjusting for the prognostic effects of *z*_*ij*_ and *y*_0*ij*_.

If a time‐to‐event outcome was of interest, then a Cox proportional hazards regression model could be fitted in each trial separately, such as

(3) $$\lambda_{\mathrm{ij}}\left(t\right)=\lambda_{0i}\left(t\right)\exp\left(\beta_{1i}z_{ij}+\beta_{2i}x_{\mathrm{ij}}+\beta_{3i}y_{0\mathrm{ij}}+\gamma_{W\;i}x_{\mathrm{ij}}z_{\mathrm{ij}}\right),$$

where *λ*_*ij*_(*t*) is the hazard rate of the outcome for the *j*th participant in the *i*th trial conditional on their covariate values, whilst *λ*_0*i*_(*t*) is the baseline hazard (ie, the hazard rate for a participant in the control group with zero values of *z*_*ij*_ and *y*_0*ij*_). Now the treatment effect is measured by a log hazard ratio, such that the treatment‐covariate interaction (*γ*_*Wi*_) represents the change in the log hazard ratio for a 1‐unit increase in *z*_*ij*_ after adjusting for the prognostic effects of *z*_*ij*_ and *y*_0*ij*_.

Extension of models 1, 2, 3 to adjust for further baseline covariates (ie, in addition to *z*_*ij*_ and *y*_0*ij*_) is recommended when there are other known prognostic factors.^19^ Ideally, the researcher should predefine a core set of strong prognostic factors to be adjusted for in each trial, particularly focusing on those routinely recorded in the trials available for IPD meta‐analysis. For example, in many disease fields, age and stage of disease are key prognostic factors and routinely recorded at baseline, such that they can be adjusted for in the analysis. Other more complex trial designs (eg, cluster trials, or multicenter or multiarm trials) would also require the models to be modified, as would allowing for nonproportional hazards in model (3).

Estimation of models 1, 2, 3 in each study, for example using (restricted) maximum likelihood estimation, produces a treatment‐covariate interaction estimate,$\;{\hat{\gamma }}_{W\;i}$, and its variance, $\mathrm{var}\left({\hat{\gamma }}_{W\;i}\right)$. The “*W*” is used to emphasize that the interaction, *γ*_*Wi*_, in each study is based solely on within‐study information; that is, it is only based on differences in the treatment effect across participant‐level covariate values observed within each study. This is an important point (as it avoids using across‐study information and potentially introducing aggregation bias), which we return to in the next section. Each *γ*_*Wi*_ indicates for trial *i* the change in treatment effect for a one‐unit increase in the participant‐level covariate *z*_*ij*_. For a continuous covariate, this assumes the effect of the interaction is linear (although extension to nonlinear trends is important, as described later in the article).

#### 3.1.2 Second stage

In the second stage of a two‐stage IPD meta‐analysis, we simply combine the ${\hat{\gamma }}_{W\;i}$ values across trials in a traditional meta‐analysis model, such as a common‐effect (sometimes known as a fixed‐effect) model,

(4) $${\hat{\gamma }}_{W\;i}∼N\left(\gamma_{W},,,\mathrm{var}\left({\hat{\gamma }}_{W\;i}\right)\right),$$

or a random‐effects model:

(5) $$\begin{array}{cc} & {\hat{\gamma }}_{W\;i}∼N\left(\gamma_{W\;i},,,\mathrm{var}\left({\hat{\gamma }}_{W\;i}\right)\right)\\ & \gamma_{W\;i}∼N\left(\gamma_{W},,,\tau^{2}\right). \end{array}$$

After estimation of the chosen meta‐analysis model (eg, using restricted maximum likelihood estimation), the estimate of *γ*_*W*_ summarizes the difference in the treatment effect for two individuals who differ in *z*_*ij*_ by one‐unit. Based on the linear, logistic, and Cox models used in the first‐stage, *γ*_*W*_ represents a difference in mean difference for a continuous outcome, a difference in log odds ratios for a binary outcome (ie, $\exp\left({\hat{\gamma }}_{W}\right)$ gives a ratio of odds ratios), and a difference in log hazard ratios for a time‐to‐event outcome (ie, $\exp\left({\hat{\gamma }}_{W}\right)$ is a ratio of hazard ratios).

Note that random‐effects meta‐analysis model (5) allows for between‐study heterogeneity in the true treatment‐covariate interaction. It may arise due to differences across studies in, for example, the dose of the treatment, the length of follow‐up, the way the covariate has been measured, and the magnitude of any interaction. It may also be due to case‐mix differences in the study populations, for example leading to between‐study differences in the distribution of within‐study confounders and even the covariate itself. For example, if a treatment‐covariate interaction is nonlinear, and the covariate distribution is narrow in some studies and wide in others, then this will induce between‐study heterogeneity in the treatment‐covariate interaction, unless the nonlinear association is modeled directly (see later). The magnitude and impact of heterogeneity can be summarized by providing estimates of *τ*^2^ and using 95% prediction intervals.^20^, ^21^ To account for uncertainty in the estimate of *τ*^2^ when deriving 95% confidence intervals for *γ*_*W*_, we recommend the Hartung‐Knapp Sidik‐Jonkman (HKSJ) approach,^22^, ^23^ or alternatively using a Bayesian framework.

If some studies do not agree to share their IPD but do provide (either directly or in a publication) the required treatment‐covariate interaction estimate and its corresponding standard error (SE), these can be incorporated in the second stage. That is, the ${\hat{\gamma }}_{W\;i}$ derived directly from IPD trials are combined with the ${\hat{\gamma }}_{W\;i}$ extracted (or provided) from non‐IPD trials.

### 3.2 The one‐stage approach

A one‐stage IPD meta‐analysis can also be used to summarize treatment‐covariate interactions. This approach analyzes the IPD from all trials in a single step using a general or generalized linear (mixed) model framework,^24^, ^25^ or a survival (frailty) model.^26^ This allows a more exact likelihood specification than that used in the second stage of the two‐stage approach, and thus avoids assuming study‐specific treatment‐covariate interaction estimates are normally distributed with known variances.^24^ Hence one‐stage models may be most advantageous when the studies in the IPD meta‐analysis have small numbers of participants and/or events.^27^, ^28^

Inclusion of a treatment‐covariate interaction term in a one‐stage model is not as straightforward as it may seem. Depending on the model specification, the apparently simple inclusion of a global treatment‐covariate interaction term may allow across‐study information to contribute toward the summary interaction estimate, in combination with within‐study information.^8^, ^29^, ^30^ This may lead to aggregation bias (also known as ecological bias), which refers to when the information across studies distorts the interaction estimate compared to when using only within‐study information.

Let ${\bar{z}}_{i}$ represent the study‐specific mean of the covariate *z*_*ij*_, and let us assume there is potential between‐study heterogeneity in the treatment‐covariate interaction. Then, to disentangle within‐study and across‐study information in a one‐stage model, there are two key options:

(i) Center the covariate *z*_*ij*_ about its study‐specific mean, ${\bar{z}}_{i}$ and add an additional term which allows the covariate means (${\bar{z}}_{i}$) to explain between‐study heterogeneity in the overall treatment effect^7^, ^31^, ^32^;

and/or.

(ii) Stratify by trial other parameters outside the interaction term, including the parameter representing the treatment effect (ie, the treatment effect at the covariate's reference value, typically *z*_*ij*_ = 0 or if the covariate is centered $z_{\mathrm{ij}}={\bar{z}}_{i}$).

Implementing either approach should give very similar summary estimates of the treatment‐covariate interaction, *γ*_*W*_, from a one‐stage IPD meta‐analysis. Approach (i) leads to a one‐stage model for continuous, binary, and time‐to‐event outcomes of the following format^7^, ^8^, ^29^:

(6) $$\begin{array}{cc} & y_{\mathrm{ij}}=\alpha_{i}+\beta_{1i}z_{\mathrm{ij}}+\beta_{2i}x_{\mathrm{ij}}+\beta_{3i}y_{0\mathrm{ij}}+\gamma_{W\;i}x_{\mathrm{ij}}\left(z_{\mathrm{ij}}-{\bar{z}}_{i}\right)+\epsilon_{\mathrm{ij}}\\ & \beta_{2i}∼N\left(\varphi +\gamma_{A}{\bar{z}}_{i},,,\tau_{1}^{2}\right)\;\gamma_{W\;i}∼N\left(\gamma_{W},,,\tau_{2}^{2}\right), \end{array}$$

(7) $$\begin{array}{cc} & y_{\mathrm{ij}}∼\text{Bernoulli}\left(p_{\mathrm{ij}}\right)\\ & \text{logit}\left(p_{\mathrm{ij}}\right)=\alpha_{i}+\beta_{1i}z_{\mathrm{ij}}+\beta_{2i}x_{\mathrm{ij}}+\beta_{3i}y_{0\mathrm{ij}}+\gamma_{W\;i}x_{\mathrm{ij}}\left(z_{\mathrm{ij}}-{\bar{z}}_{i}\right)\\ & \beta_{2i}∼N\left(\varphi +\gamma_{A}{\bar{z}}_{i},,,\tau_{1}^{2}\right)\;\gamma_{W\;i}∼N\left(\gamma_{W},,,\tau_{2}^{2}\right), \end{array}$$

(8) $$\begin{array}{cc} & \lambda_{\mathrm{ij}}\left(t\right)=\lambda_{0i}\left(t\right)\exp\left(\beta_{1i}z_{\mathrm{ij}}+\beta_{2i}x_{\mathrm{ij}}+\beta_{3i}y_{0\mathrm{ij}}+\gamma_{W\;i}x_{\mathrm{ij}}\left(z_{\mathrm{ij}}-{\bar{z}}_{i}\right)\right)\\ & \beta_{2i}∼N\left(\varphi +\gamma_{A}{\bar{z}}_{i},,,\tau_{1}^{2}\right)\;\gamma_{W\;i}∼N\left(\gamma_{W},,,\tau_{2}^{2}\right). \end{array}$$

Parameters in models 6, 7, 8 are similar to those defined for models 1, 2, 3 in Section 3.1.1. In addition, the study‐specific treatment‐covariate interactions (*γ*_*Wi*_) are assumed normally distributed with a mean *γ*_*W*_ and between‐study variance $\tau_{2}^{2}$, as for random‐effects model (5) in the second stage of the two‐stage approach. Of key interest is an estimate of *γ*_*W*_, to summarize the expected change in the treatment effect (eg, log hazard ratio in model (8)) for each one unit increase in *z*_*ij*_. As previously, “*W*” indicates that the interaction will be based solely on within‐study information.

Models 6, 7, 8 also include a meta‐regression component ($\varphi +\gamma_{A}{\bar{z}}_{i})$, where *φ* denotes the summary treatment effect for participants with zero values of *z*_*ij*_ and *y*_0*ij*_ in trials with ${\bar{z}}_{i}$ equal to zero; and *γ*_*A*_ is the change in the summary treatment effect for each 1‐unit increase in ${\bar{z}}_{i}$. The models also allow for residual between‐study variance ($\tau_{1}^{2}$) in the summary treatment effect (ie, that not explained by ${\bar{z}}_{i}$). Inclusion of the $\gamma_{A}{\bar{z}}_{i}$ term, together with the centering of *z*_*ij*_ within the interaction term, disentangles *γ*_*W*_ and *γ*_*A*_ such that they are uncorrelated with each other, and thus ${\hat{\gamma }}_{W}$ will be based solely on within‐trial information.^8^, ^32^ Note that if the $\gamma_{A}{\bar{z}}_{i}$ term is not included in model (8), then the interaction term will represent some weighted average of ${\hat{\gamma }}_{W}$ and the magnitude of aggregation bias (${\hat{\gamma }}_{E}$ = ${\hat{\gamma }}_{A}-{\hat{\gamma }}_{W}$). Other parameters (ie, intercepts, baseline hazards, prognostic effects of *z*_*ij*_ and *y*_0*ij*_) are stratified by trial (ie, estimated separately for each trial).

Approach (ii) leads to one‐stage models for continuous, binary and time‐to‐event outcomes of the following format:

(9) $$\begin{array}{cc} & y_{\mathrm{ij}}=\alpha_{i}+\beta_{1i}z_{\mathrm{ij}}+\beta_{2i}x_{\mathrm{ij}}+\beta_{3i}y_{0\mathrm{ij}}+\gamma_{W\;i}x_{\mathrm{ij}}z_{\mathrm{ij}}+\epsilon_{\mathrm{ij}}\\ & \gamma_{W\;i}∼N\left(\gamma_{W},,,\tau^{2}\right), \end{array}$$

(10) $$\begin{array}{cc} & y_{\mathrm{ij}}∼\text{Bernoulli}\left(p_{\mathrm{ij}}\right)\\ & \text{logit}\left(p_{\mathrm{ij}}\right)=\alpha_{i}+\beta_{1i}z_{\mathrm{ij}}+\beta_{2i}x_{\mathrm{ij}}+\beta_{3i}y_{0\mathrm{ij}}+\gamma_{W\;i}x_{\mathrm{ij}}z_{\mathrm{ij}}\\ & \gamma_{W\;i}∼N\left(\gamma_{W},,,\tau^{2}\right), \end{array}$$

(11) $$\begin{array}{cc} & \lambda_{\mathrm{ij}}\left(t\right)=\lambda_{0i}\left(t\right)\exp\left(\beta_{1i}z_{\mathrm{ij}}+\beta_{2i}x_{\mathrm{ij}}+\beta_{3i}y_{0\mathrm{ij}}+\gamma_{W\;i}x_{\mathrm{ij}}z_{\mathrm{ij}}\right)\\ & \gamma_{W\;i}∼N\left(\gamma_{W},,,\tau^{2}\right). \end{array}$$

In models 9, 10, 11 each of the “nuisance” parameters (ie, the *α*_*i*_, *λ*_0*i*_(*t*), *β*_1*i*_, *β*_2*i*_, and *β*_3*i*_ parameters which are not of primary interest) are stratified by trial, and random effects are placed only on the within‐study interaction. This stratification of all nuisance parameters by trial, in particular *β*_2*i*_ representing the treatment effect at the covariate's reference value, ensures that *γ*_*W*_ only contains within‐trial information. It also closely reflects the two‐stage approach, where all nuisance parameters are naturally stratified by trial as they are estimated in each trial separately in the first stage.

As mentioned for the two‐stage approach, all these one‐stage models 6, 7, 8, 9, 10, 11 need to be modified for other trial designs (eg, cluster trials) and when adjusting for further prognostic factors (ie, in addition to *z*_*ij*_ and *y*_0*ij*_). The latter is a sensible strategy (see discussion in Section 3.1.1), but again requires each prognostic factor's effect to be stratified by study. This will increase the number of parameters and so, for estimation reasons, centering each included prognostic factor by its study‐specific mean is a sensible default approach (especially when unrestricted maximum likelihood estimation is used to fit the one‐stage model).

### 3.3 Applied example: Is the effect of antihypertensive treatment different for males and females?

The two‐stage and one‐stage approaches are now illustrated using the IPD from the 10 randomized trials examining the effect of antihypertensive treatment on SBP, as introduced in Section 2.1. The question is whether the treatment effect is different for males compared to females; that is, whether there is a treatment‐sex interaction.

*Two‐stage approach*

**Figure 1:**
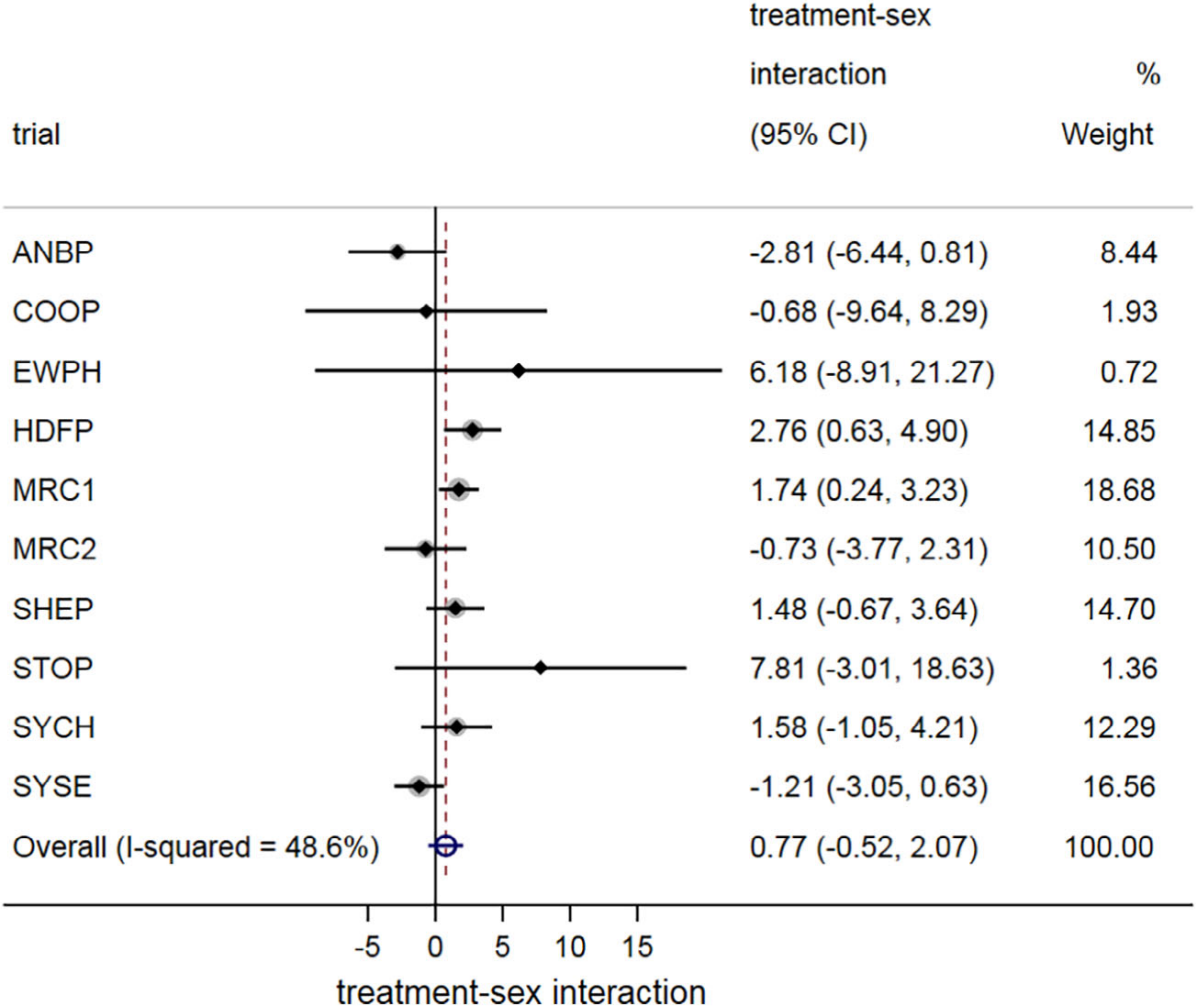
A two‐stage IPD meta‐analysis of treatment‐sex interactions, summarizing the difference in the effect of antihypertensive treatment for males compared to females. *Note*: The interactions refer to the difference between males and females in the treatment effect (ie, their difference in the mean difference in final systolic blood pressure for treatment vs control, after adjusting for baseline), with negative values indicating that the treatment effect is better in females than males [Colour figure can be viewed at wileyonlinelibrary.com]

In the first stage, restricted maximum likelihood (REML) estimation was used to fit model (1) with final SBP as the response, and sex (males = 1, females = 0) as the covariate, *z*_*ij*_, of interest. Figure 1 provides a forest plot of the treatment‐sex interaction estimates, ${\hat{\gamma }}_{W\;i}$, plotted as circles. Circles are recommended by Fisher et al to help distinguish a forest plot of interaction estimates from a standard forest plot of treatment effect estimates,^6^, ^9^, ^33^ for which squares are typically used. In the second stage, REML estimation was used to fit random‐effects meta‐analysis model (5) and this gave a summary interaction estimate of ${\hat{\gamma }}_{W}$ = 0.77 (95% CI: −0.52 to 2.07). Hence there is no evidence of an important difference in treatment effect for males compared to females; the summary interaction estimate is close to zero and clinically unimportant, the confidence interval overlaps zero, and there is also between‐study heterogeneity. Using the HKSJ approach, the 95% confidence is slightly wider (−0.73 to 2.27).

*One‐stage approach*

For the one‐stage approach, we used REML estimation to fit models (6) and (9). This gave the same summary interaction estimate of ${\hat{\gamma }}_{W}$ = 0.77 (95% CI: −0.52 to 2.06), practically identical to that from the two‐stage approach when REML and a Wald‐based confidence interval are used. When we rather used the Satterthwaite approach to derive confidence intervals following estimation of the one‐stage model (as recommended for continuous outcomes^34^, ^35^) the obtained 95% confidence interval for *γ*_*W*_ was slightly wider (−0.72 to 2.26), and practically identical to that derived using the HKSJ method in the two‐stage approach (as they both utilize the *t*‐distribution rather than the standard normal distribution). Thus, one‐stage and two‐stage IPD meta‐analysis results for the treatment‐sex interaction are almost identical. This agrees with Burke et al who describe in detail that when the same assumptions and the same estimation approaches are applied, one‐stage and two‐stage approaches will closely agree unless most studies are small.^28^ In conclusion, this example does not support there being an important treatment‐sex interaction.

## 4 STATISTICAL MODELING RECOMMENDATIONS WHEN CONDUCTING AN IPD META‐ANALYSIS TO EXAMINE TREATMENT‐COVARIATE INTERACTIONS

Some published IPD meta‐analysis projects that examine treatment‐covariate interactions use inappropriate statistical approaches that ignore or incorrectly modify the models described in Section 3.^9^ We now make five recommendations to address the key concerns.

### 4.1 Do not make inferences about interactions using the summary treatment effect derived in each subgroup separately

When the participant‐level covariate is categorical, it may be tempting to perform a one‐stage or two‐stage IPD meta‐analysis of the overall treatment effect in each category (subgroup) separately. For example, an IPD meta‐analysis might be conducted for males and females separately, to obtain the summary treatment effect for males and females. However, Fisher et al and Belias et al show that it is dangerous to use the subsequent results to make inferences about whether an interaction exists.^9^, ^36^ In particular, a common mistake is to conclude a treatment‐covariate interaction exists if the summary treatment effect estimate is statistically significant in one subgroup but not the other. In this situation the actual treatment‐covariate interaction (difference between subgroups based on within‐study information) may not be statistically significant. Altman and Bland consider this eloquently.^37^

It is even flawed to compare the summary treatment effects for each subgroup, for example via a statistical test or by calculating their difference. Although this is apparently simple, it amalgamates within‐study and across‐study information, and so should be avoided. The larger the differences in mean covariate values across studies, the larger the potential contribution of the across‐study information. One way to understand this is to consider an extreme situation, where a treatment‐sex interaction is of interest, but some trials contain only males. Such trials cannot contribute any within‐study information about the interaction between treatment effect and sex at the individual level, as there are no females. However, the trial would still contribute toward the subgroup result for males, and thus subsequently toward the difference between meta‐analysis results for male and female subgroups. This issue is avoided by meta‐analyzing the treatment‐covariate interaction estimates observed within trials, by using the two‐stage and one‐stage approaches outlined in Section 3.

*Application to the hypertension example*

Recall that a two‐stage IPD meta‐analysis using only within‐study information gave a summary treatment‐sex interaction estimate of ${\hat{\gamma }}_{W}$ = 0.77 (95% CI: −0.52 to 2.07). When we do a separate IPD meta‐analysis for males and females, we obtain summary treatment effect estimates of −9.02 (95% CI: −10.47 to −7.57) and −10.59 (95% CI: −12.60 to −8.58), respectively. The estimated difference in these subgroup results is 1.57, which is an amalgamation of within‐trial and across‐trial information, and about twice the size of the summary interaction estimate of 0.77 based solely on within‐study information. The across‐trial information arises because the proportion of males varies considerably across trials, from about 0.23 to 0.70.

### 4.2 Separate within‐trial and across‐trial information in one‐stage models

One‐stage IPD meta‐analysis models are appealing to statisticians, as they eloquently synthesize all the IPD in a single statistical model as described in Section 3.2. However, compared to the two‐stage approach, it is easier to make modeling errors when estimating *γ*_*WA*_. One‐stage models 6, 7, 8, 9, 10, 11 show how to separate within‐study and across‐study information, and ensure that *γ*_*WA*_ is estimated based solely on within‐study information. As such, they are labeled as “deft” by Fisher et al.^9^ However, many researchers wrongly fit models 6, 7, 8 without the $\gamma_{A}{\bar{z}}_{i}$ term, and with $\gamma_{W\;i}x_{\mathrm{ij}}\left(z_{\mathrm{ij}}-{\bar{z}}_{i}\right)$ replaced by *γ*_*WAi*_*x*_*ij*_*z*_*ij*_. For example, a flawed one‐stage linear regression model for a continuous outcome is:

(12) $$\begin{array}{cc} & y_{\mathrm{ij}}=\alpha_{i}+\beta_{1i}z_{\mathrm{ij}}+\beta_{2i}x_{\mathrm{ij}}+\beta_{3i}y_{0\mathrm{ij}}+\gamma_{\mathrm{WAi}}x_{\mathrm{ij}}z_{\mathrm{ij}}+e_{\mathrm{ij}}\gamma_{\mathrm{WAi}}∼N\left(\gamma_{\mathrm{WA}},,,\tau_{\gamma_{\mathrm{WA}}}^{2}\right)\\ & \beta_{2i}∼N\left(\beta_{2},,,\tau_{\beta_{2}}^{2}\right)\;e_{\mathrm{ij}}∼N\left(0,,,\sigma_{i}^{2}\right). \end{array}$$

Here, the interaction *γ*_*WA*_ is an amalgamation of within‐study information and across‐study information; essentially a weighted average of *γ*_*W*_ and *γ*_*A*_.^8^ The estimate of *γ*_*WA*_ from model (12) will be more precise than the estimate of *γ*_*W*_ from model (6), but at the expense of the across‐study information distorting the estimate and interpretation compared to *γ*_*W*_. This motivates Fisher et al to call it a “deluded” analysis.^9^

*Application to the hypertension example*

**Figure 2:**
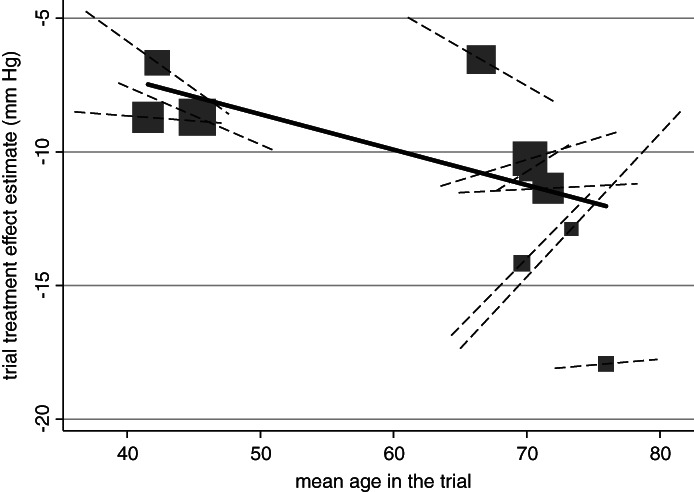
Does age interact with the effect of antihypertensive treatment on systolic blood pressure? Findings from an IPD meta‐analysis of 10 trials, showing difference in results based on across‐study (solid line) and within‐study (dashed lines) information. Across‐study relationship (from meta‐regression of trial's treatment effect estimates vs mean age) denoted by gradient of solid line. Participant‐level relationship using within‐study information (ie, treatment‐sex interaction within each trial) denoted by gradient of dashed lines and the summary gradient (${\hat{\gamma }}_{W})$ is −0.036 (95% CI: −0.19 to 0.12). Each block represents one trial, and the block size is proportional to the size of the trial.

Returning to the hypertension example, let us consider whether there is a treatment‐age interaction whilst assuming that the effect of age is linear. REML estimation of one‐stage model (6) gives ${\hat{\gamma }}_{W}=$−0.036 (95% CI: −0.19 to 0.12), and thus no clear evidence of a treatment‐age interaction. When rather fitting model (12), we obtain ${\hat{\gamma }}_{\mathrm{WA}}$= −0.079 (95% CI: −0.14 to −0.02), which is almost twice the size of that based solely on within‐study information and now strongly suggests a treatment‐age interaction. The difference is because ${\hat{\gamma }}_{\mathrm{WA}}$ is strongly influenced by the across‐study information, which is suggesting a different magnitude of effect than suggested by the within‐study information in half of the trials. This is illustrated in Figure 2, which also suggests that there may be a nonlinear interaction with age, as the direction of the interactions appears to be different in trials with younger and older ages. Extension to nonlinear trends is considered later.

### 4.3 Do not dichotomize continuous covariates or outcomes

Categorization, and in particular dichotomization, of continuous covariates (eg, such as age, blood pressure, and most biomarkers) using cut‐points is best avoided when examining their interaction with treatment effect.^38^, ^39^, ^40^ The usual argument for categorization is to aid clinical interpretation and maintain simplicity. However it can rarely, if ever, be justified that an individual whose value is just below the cut‐point is completely different from an individual whose value is just above it. Moreover, categorization reduces the power to detect genuine covariates that interact with treatment effect. Ensor et al show that, in the aforementioned IPD meta‐analysis to examine exercise interventions to reduce gestational weight gain in pregnancy, the loss of information by dichotomizing BMI (rather than keeping BMI as continuous) is equivalent to throwing away about one‐third of the IPD available.^41^

Categorization of continuous *outcomes* should also be avoided, as it can mislead researchers into thinking there are differential responses to treatment. In particular, classifying individuals as either responders or nonresponders, based on an arbitrary cut‐point value for a continuous outcome (eg, SBP < 120 mmHg at follow‐up), will lead to those just above the threshold being classed differently to those just below it, which is nonsense. Such dichotomization also leads to misclassification when there is measurement error.^42^

*Application to the pregnancy example*

In the pregnancy example an outcome of interest was caesarean section and, across all participants in 32 trials, the intervention reduces the odds of caesarean section by about 10% (summary OR = 0.91; 95% CI: 0.83 to 0.99). It is of interest whether the mother's age interacts with this intervention effect. If we arbitrarily dichotomize age at 35 years, so that it becomes a binary variable (ie, 0 if age ≤35, and 1 if age >35), then a two‐stage IPD meta‐analysis (model (2) followed by model (5)) shows no clear evidence of an interaction between age and treatment effect (summary ratio of ORs = 1.14, 95% CI: 0.90 to 1.45). However, if age is kept as a continuous variable, then there is stronger evidence of a treatment‐age interaction. The intervention becomes less effective at reducing the odds of caesarean section as a women's age increases. For every 5‐year increase in age the odds ratio increases by about 10% (summary ratio of ORs = 1.10, 95% CI: 1.00 to 1.20). When analyzed on the risk (rather than odds scale), using a binomial regression with a log‐link, this translates to about a 6% increase in the risk ratio for every 5‐year increase (95% CI: 0.98 to 1.15).

### 4.4 Allow for potential nonlinear relationships when modeling interactions with continuous covariates

In the previous example we assumed a linear trend for the interaction of treatment and a continuous covariate. However, sometimes the interaction may be nonlinear, as emphasized by Royston and Sauerbrei,^15^ and considered in detail by Kasenda et al.^43^, ^44^ This implies the change in treatment effect for every 1‐unit increase in the covariate may vary across the distribution of the covariate. Therefore, nonlinear interactions should be evaluated when the interaction of a continuous covariate and treatment effect is of interest. We now illustrate this with an example, and explain how it can be implemented using a multivariate meta‐analysis of spline functions.

*Application to the hypertension example*

**Figure 3:**
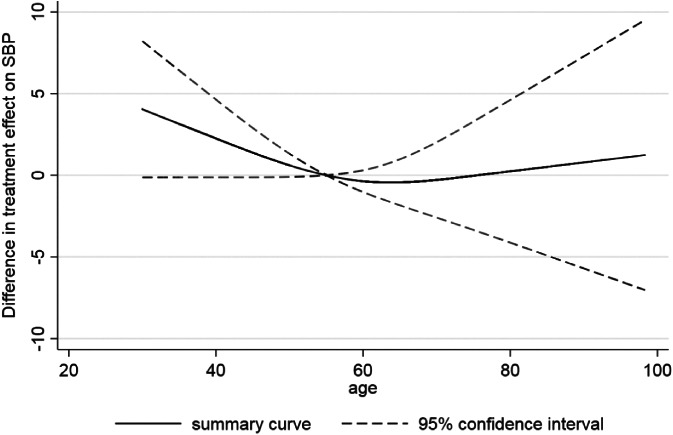
Summary of the nonlinear interaction between age and effect of hypertension treatment on final SBP value. Figure created by fitting an analysis of covariance model in each study separately, with the interaction between age and treatment modeled via a restricted cubic spline function (with knot positions of 39, 60, and 75), and then the study‐specific parameter estimates (relating to the interaction) pooled in a multivariate random‐effects meta‐analysis

Wang et al consider whether there is an interaction between age and the effect of hypertensive treatment, and identify a nonlinear relationship, with older patients between 60 and 80 years old seeming to benefit more than younger patients and those older than 80.^17^ Their original analysis categorized age, but we now update their analysis to rather display the change in treatment effect as a smooth, nonlinear function of age (Figure 3), with the reference group being the treatment effect for a 55 year old. A J‐shaped relationship is visible; in particular, there is strong evidence that younger individuals have the smallest treatment effect. For example, compared to an individual aged 40 years, an individual aged 55 years has about a 3 mmHg greater reduction in SBP due to the treatment (compared to control). In very old ages the treatment effect also appears to reduce slightly, but there is large uncertainty (wide confidence intervals). Interestingly, if we just include an interaction with age (ie, assume a linear interaction term) then there is no evidence of a treatment‐age interaction. The change in treatment effect for each year increase of age is −0.05 (95% CI: −0.19 to 0.12). The linear assumption hides the more J‐shaped interaction revealed by the nonlinear modeling approach.

*Conducting a two‐stage multivariate IPD meta‐analysis of restricted cubic splines*

**Figure 4:**
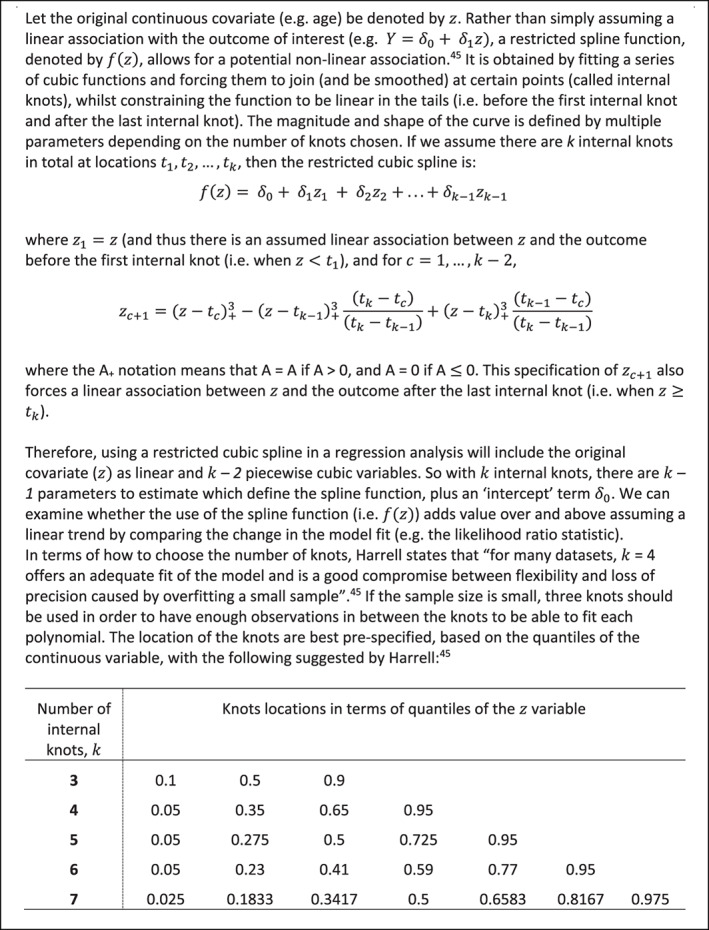
Introduction to modeling nonlinear relationships in a single study using restricted cubic splines; for further details we recommend the reader refer to Harrell^45^

Figure 3 was obtained by conducting a two‐stage IPD meta‐analysis, where in the first stage a restricted cubic spline function was estimated in each study separately and in the second stage a multivariate meta‐analysis was fitted. Splines are a flexible way of modeling smooth nonlinear relationships.^45^ Briefly, a restricted cubic spline is obtained by fitting a series of cubic functions and forcing them to join (and be smoothed) at certain points (called internal knots), whilst constraining the function to be linear in the tails (ie, before the first internal knot and after the last internal knot). The magnitude and shape of the curve are defined by multiple parameters depending on the number of knots chosen; as explained in Figure 4. The knot locations are forced to be the same in every study, to ensure the study‐specific curves can be synthesized meaningfully. Rather than using a reference group whose covariate value is 0, it helps to center the covariate at a meaningful value (eg, 55 years was chosen as the reference group in Figure 3), which is the same in every study. In our example, three internal knots (at the same locations in every study) were chosen for the restricted cubic function representing the association between age and final SBP in the control group. Hence, in the first stage three parameters defining the spline function are estimated in each study (an intercept and two slope terms), plus the interaction of this spline function with the treatment effect. The latter provides the within‐study treatment‐covariate interaction defined by *γ*_*W*1*i*_ and *γ*_*W*2*i*_ (see Figure 5A), which represent the change (difference) in treatment effect across covariate values, relative to the chosen reference group of an individual aged 55 years.

**Figure 5:**
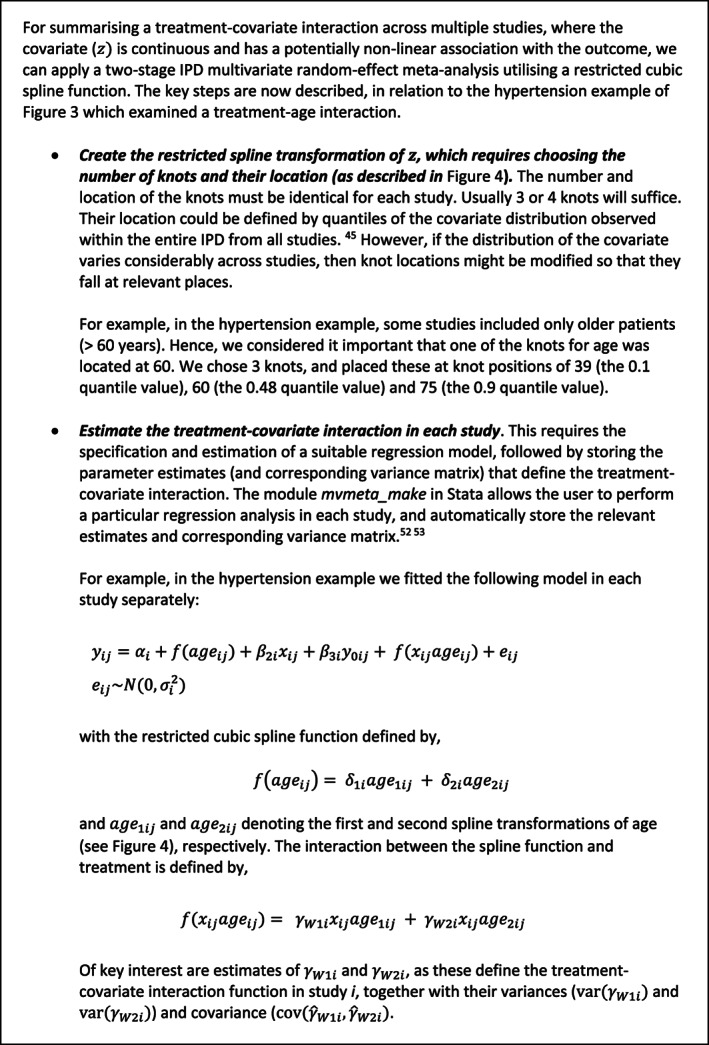
(A): Overview of the first stage of a two‐stage multivariate IPD meta‐analysis to summarize a nonlinear treatment‐covariate interaction using a restricted cubic spline. The steps are described in relation to the hypertension example of Figure 3 which examined a treatment‐age interaction.

**Figure 5:**
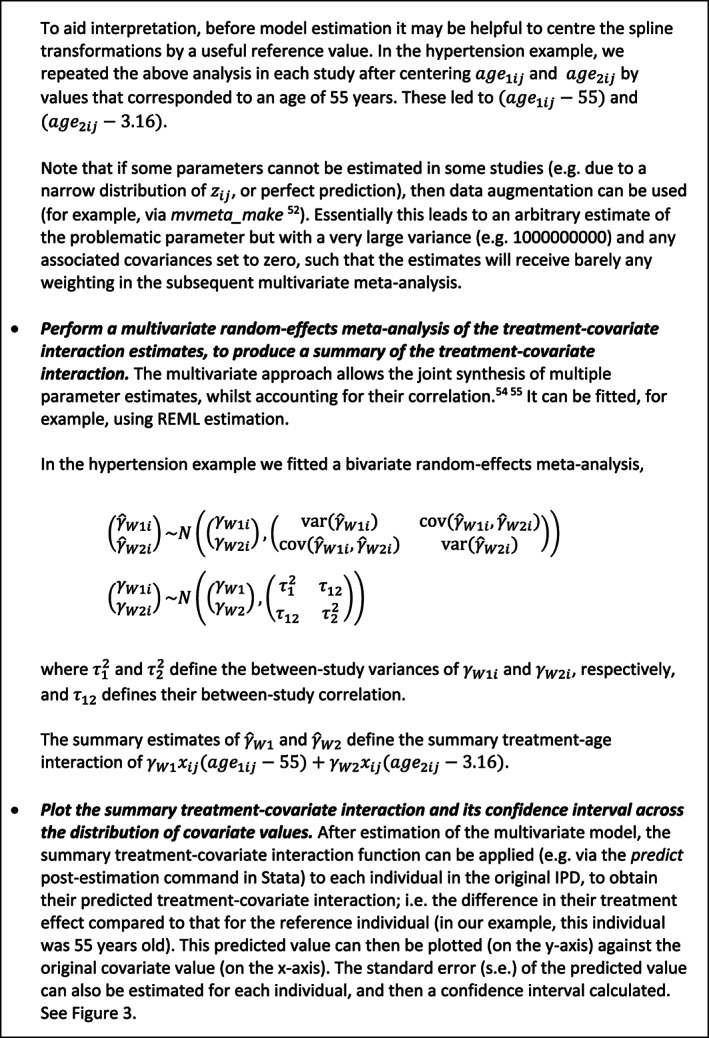
(B): Overview of the second stage of a two‐stage multivariate IPD meta‐analysis to summarize a nonlinear treatment‐covariate interaction using a restricted cubic spline. The steps are described in relation to the hypertension example of Figure 3 which examined a treatment‐age interaction

In the second stage, the study estimates of the difference in slope parameters of the spline function (ie, ${\hat{\gamma }}_{W1i}$ and ${\hat{\gamma }}_{W2i}$) can be meta‐analyzed using a multivariate random‐effects meta‐analysis.^46^, ^47^, ^48^, ^49^, ^50^ This allows the synthesis of multiple parameter estimates whilst accounting for their within‐study and between‐study correlation, and produces a summary estimate for each parameter, from which the summary spline (nonlinear) function can be derived. It can handle missing parameter estimates (ie, for missing parts of the spline function in studies with a narrow distribution of covariate values) as described in Figure 5A,B. This summary curve describes the association between values of the covariate and the change in treatment effect, relative to the reference group (age 55 years in the example). This can then be plotted graphically to aid interpretation. The study‐specific estimated curves from the first stage (or study‐specific empirical Bayes curves obtained postestimation from the second stage) might also be presented, as shown by Gasparrini et al.^46^

A summary of the two‐stage multivariate IPD meta‐analysis of restricted spline functions is given in Figure 5A,B. A similar approach is a multivariate IPD meta‐analysis of a polynomial function^51^; in the first stage the differences (ie, treatment minus control) of the parameters of a chosen polynomial function are estimated in each study (eg, differences in a quadratic shape, such as $\gamma_{W1}x_{\mathrm{ij}}z_{\mathrm{ij}}+\gamma_{W2}x_{\mathrm{ij}}z_{\mathrm{ij}}^{2}$) and the parameter estimates (${\hat{\gamma }}_{W1}$ and ${\hat{\gamma }}_{W2}$) are jointly synthesized to produce a summary function.^51^ In particular, fractional polynomial functions provide a flexible set of power transformations to describe a potentially nonlinear association.^52^, ^53^, ^54^ In order for the parameters of the function to be combinable across studies in a multivariate meta‐analysis, the same powers of the fractional polynomial function must be specified in each study (ie, the shape of the nonlinear association is fixed across studies).^51^ In contrast, as long as the same number and location of knots is used in each study, a restricted cubic spline function is more flexible (ie, the shape of the nonlinear association can vary across studies) and it can handle different covariate distributions across studies by strategic placement of knots. Sauerbrei and Royston show that it is possible to combine different fractional polynomial power transformations across studies, if the predicted values of the function are pooled (rather than the parameters defining the function),^55^ with the pooling of predictions done at each value of the covariate separately.

A one‐stage IPD meta‐analysis model can also be used to examine nonlinear treatment‐covariate interactions, but difficulties can arise. First, the extra parameters required to model nonlinear functions, and potentially multiple random effects, may cause estimation and convergence problems. Second, when centering covariates by their study‐specific means (ie, to avoid aggregation bias), by extending models 6, 7, 8, the interpretation of the spline function becomes problematic. Unless all studies have the same mean covariate value, the change in treatment effect for a 1‐unit increase in a covariate from its mean will have a different interpretation in each study; this will make the summary spline function uninterpretable. Therefore, it is preferable to examine nonlinear trends by extending one‐stage models 9, 10, 11, stratifying by trial parameters outside the interaction term, to remove aggregation bias.

### 4.5 To personalize decision‐making, individualized predictions of treatment effect are required

For clinical practice, translation of identified treatment‐covariate interactions is required for the individual patient, in order to tailor their treatment decisions. This is nontrivial. A fundamental error is to assume that if a treatment‐covariate interaction exists then the treatment is effective in some individuals but not in others. Actually, even when the magnitude of treatment effect varies across individuals, the direction of effect may consistently suggest the treatment is beneficial for everyone.

To better guide decision‐making, a predicted treatment effect is required for each individual. This requires a robust prediction model equation, developed and validated using methodology principles for clinical prediction models.^2^, ^45^, ^56^ In particular, to reduce overfitting (too extreme predictions) the model development might require penalization and shrinkage approaches,^57^, ^58^, ^59^ to reduce the variability of predictions in new datasets, thereby reducing the mean‐square error of the predictions. A step further is to predict an individual's absolute outcome value or risk conditional on their prognostic factors and expected treatment effect. These and related issues are discussed in detail elsewhere.^60^, ^61^, ^62^, ^63^ Crucially, aggregation bias should also be avoided when making predictions, and we now illustrate this with an example.

*Application to the hypertension example*

Consider the example within Figure 3 that suggests a potential nonlinear interaction between age and the effect of antihypertensive treatment, with younger patients seemingly having less benefit (in terms of reduction in SBP) than older individuals. The smooth curve reflects the treatment‐age interaction; it reveals the summary estimate of the *difference* in treatment effect for an individual with a particular age compared to an individual aged 55 years. However, it does *not* tell us the predicted (expected) treatment effect for an individual with a particular age. To obtain this, we performed a multivariate IPD random‐effects meta‐analysis, where we estimate and then meta‐analyze the two slopes (*γ*_*W*1*i*_ and *γ*_*W*2*i*_) of the spline function (as defined in Figure 5A), and also the reference treatment group (*β*_2*i*_, the study‐specific treatment effect for individuals aged 55 years, our reference group). Then, the summary estimates (${\hat{\beta }}_{2},{\hat{\gamma }}_{W1},{\hat{\gamma }}_{W2})$ obtained were used to define the prediction model for an individual's treatment effect conditional on their age:

(13) $$\begin{array}{cc} \text{Predicted treatment effect for}\;j\mathrm{th}\;\text{individual} & ={\hat{\beta }}_{2}+{\hat{\gamma }}_{W1}\left({\mathrm{age}}_{1j}-55\right)+{\hat{\gamma }}_{W2}\left({\mathrm{age}}_{2j}-3.16\right)\\ & \;=-10.66\text{–}\left(0.251\times \left({\mathrm{age}}_{1j}-55\right)\right)\\ & \;+\left(0.156\times \left({\mathrm{age}}_{2j}-3.16\right)\right). \end{array}$$

Here, −10.66 is the predicted treatment effect for an individual aged 55 years, and age_1*j*_ and age_2*j*_ represent, respectively, the individual's values of the first and second spline transformation of their age. For example, for an individual aged 40, their corresponding age_1*j*_ is 40 and age_2*j*_ is 0.00077, and thus their predicted treatment effect is

$$-10.66\text{–}\left(0.251\times \left(40\text{–}55\right)\right)+\left(0.15\times \left(0.00077\text{–}3.16\right)\right)=-7.39.$$

**Figure 6:**
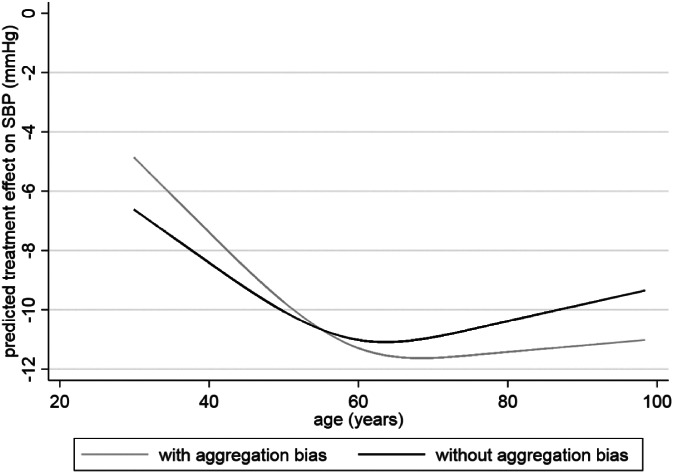
The predicted effect of antihypertensive treatment on SBP conditional on an individual's age, based on a multivariate meta‐analysis either with or without aggregation bias in the summary treatment‐age interactions

Overfitting is potentially of limited concern for prediction Equation (13), as it was derived using over 28 000 participants with only two parameters (excluding the intercept) estimated. The predicted treatment effect across the age range is summarized in Figure 6. Across all ages the predicted treatment effect is at least 5 mmHg. Hence, on average, all patients are predicted to benefit by a clinically useful amount, even though the magnitude of effect varies due to the treatment‐age interaction.

A concern is that a multivariate meta‐analysis of ${\hat{\beta }}_{2i},{\hat{\gamma }}_{W1i},$ and ${\hat{\gamma }}_{W2i}$ accounts for the correlation amongst these parameters, both within and between studies. Yet again this allows the potential for aggregation bias to influence the summary interaction terms, ${\hat{\gamma }}_{W1}$ and ${\hat{\gamma }}_{W2}$, as their correlation with ${\hat{\beta }}_{2}$ allows the borrowing of across‐trial information. Though it is perhaps sensible for ${\hat{\beta }}_{2}$ (as it is our reference treatment effect averaged across studies), we should want our summary interactions terms to be based solely on within‐trial information, as previously argued. To address this, we replace the summary ${\hat{\gamma }}_{W1}$ and ${\hat{\gamma }}_{W2}$ estimates in our prediction Equation (13) with −0.178 and 0.133, their respective estimates from a multivariate meta‐analysis ignoring any correlation with ${\hat{\beta }}_{2i}$ terms Then, the modified prediction equation is:

(14) $$\begin{array}{cc} \text{Predicted treatment effect for}\;j\mathrm{th}\;\text{individual} & ={\hat{\beta }}_{2}+{\hat{\gamma }}_{W1}\left({\mathrm{age}}_{1j}-55\right)+{\hat{\gamma }}_{W2}\left({\mathrm{age}}_{2j}-3.16\right)\\ & \;=-10.66\text{–}\left(0.178\times \left({\mathrm{age}}_{1j}-55\right)\right)\\ & \;+\left(0.133\times \left({\mathrm{age}}_{2j}-3.16\right)\right). \end{array}$$

This allows us to produce predicted treatment effects conditional on age, summarized across all studies and removing aggregation bias. Using Equation (14) for an individual aged 40 years, their predicted treatment effect is,

$$-10.66\text{–}\left(0.178\times \left(40\text{–}55\right)\right)+\left(0.133\times \left(0.00077\text{–}3.16\right)\right)=-8.41\;\text{mmHg}$$

which is larger than the −7.39 predicted from the previous equation. Indeed, the predicted curve across the entire age range is noticeably shallower after removing aggregation bias (Figure 6), because the aggregation bias (arising from incorporating across trial information) makes interaction estimates larger than when based solely on within‐trial information. Note that the predictions shown here are averaged across studies. If possible, incorporation of study‐level covariates (eg, country) that explain between‐study heterogeneity in parameter estimates might help tailor predictions further.

## 5 TO IPD OR NOT TO IPD? STATISTICAL RECOMMENDATIONS WHEN PLANNING AN IPD META‐ANALYSIS PROJECT TO EXAMINE TREATMENT‐COVARIATE INTERACTIONS

Statistical considerations are also important when deciding whether to initiate an IPD meta‐analysis project (ie, before IPD collection), and two key issues are now discussed in the context of treatment‐covariate interactions.

### 5.1 The decision to initiate an IPD meta‐analysis project to examine treatment‐covariate interactions should not be based on the amount of between‐study heterogeneity in the overall treatment effect

Without IPD, most meta‐analyses of randomized trials will summarize the treatment effect based on all trial participants (ie, without consideration of treatment‐covariate interactions). If such a meta‐analysis does not find evidence of between‐study heterogeneity in the treatment effect, it may be tempting to conclude that the treatment effect is the same for all individuals. However, this logic is flawed: the absence of heterogeneity in the overall effect (across all participants) does not necessarily imply an interaction does not exist. First, if there is a genuine treatment‐covariate interaction, but the distribution of the covariate is very similar across studies, then (assuming no other patient‐level or study‐level effect modifiers differ across studies), the overall treatment effect will be the same in each study (ie, there will be no heterogeneity). Second, even if the distribution of the covariate does change across studies, the overall treatment effect may still be homogenous; for example this may arise due to chance, or because the covariate has a nonlinear (eg, U‐shaped) interaction with treatment (so that the overall treatment effect may still be the same in two studies with very different mean covariate values), or even due to multiple effect modifiers acting in combination and different directions.

Conversely, if there is heterogeneity in the overall treatment effect this does not imply that an interaction exists at the participant‐level. Heterogeneity can arise due to changes in study‐level characteristics such as dose, follow‐up length, and setting, even when there is no interaction at the participant‐level. Hence, the decision to investigate treatment‐covariate interactions should not be driven by the presence of between‐study heterogeneity in treatment effect, and should be rather motivated by the supposed (biological) mechanism of treatment response.

*Application to the hypertension example*

**Figure 7:**
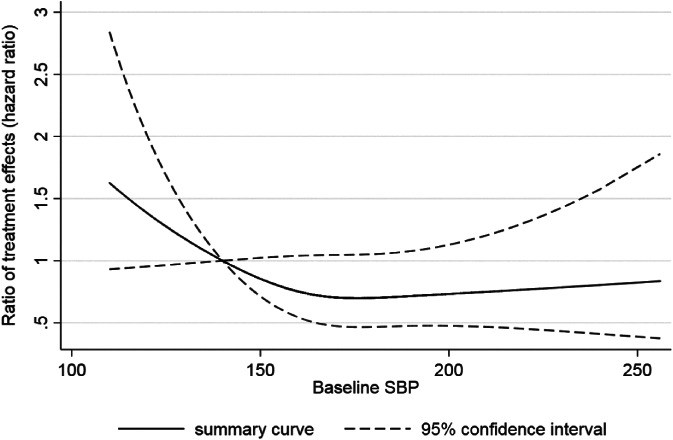
Evidence of a potential nonlinear interaction between baseline SBP and the effect of hypertension treatment on the rate of CVD, even though there was no between‐study heterogeneity in the overall treatment effect

As an illustration of why heterogeneity is a poor indicator of whether treatment‐covariate interactions exist, consider a two‐stage IPD meta‐analysis examining the effect of antihypertensive treatment on rate of CVD, for which the summary hazard ratio was 0.74 (95% CI: 0.67 to 0.81) with no observed heterogeneity (${\hat{\tau }}^{2}=0$).^64^ However, there is some suggestion of a nonlinear interaction between baseline SBP and the treatment effect on CVD, with the treatment effect gradually reducing as the baseline SBP moves from about 170 mmHg toward 120 mmHG (Figure 7), as obtained using a two‐stage multivariate meta‐analysis of restricted cubic splines as described in Figure 5A,B.

### 5.2 Calculate the power to identify a treatment‐covariate interaction prior to collection of IPD

IPD meta‐analysis projects are more likely to be funded if they have sufficient power to answer the research question at hand. Before IPD collection, power calculations for IPD meta‐analysis projects can be made conditional on the number of trials promising their IPD, using known trial characteristics such as the number of participants and standard deviation (SD) of covariate values. Closed‐form solutions are difficult to obtain, especially for noncontinuous outcomes unless approximations are made.^65^ A pragmatic starting point is to consider whether the total sample size (and overall outcome proportion or rate for binary and time‐to‐event outcomes) of the promised IPD has adequate power if naively considered to come from a single study. Then standard statistical software for estimating the power of an interaction in a single randomized trial could be used.

To mirror the IPD meta‐analysis setting more exactly, a simulation‐based approach has been proposed.^66^, ^67^, ^68^, ^69^ In particular, Ensor et al suggest generating IPD containing the same number of studies (and same number of participants and events therein) as promising their IPD,^41^ with covariate values simulated based on assumed true treatment effects and treatment‐covariate interaction effect size. Then a two‐stage IPD meta‐analysis is applied to this simulated dataset, and the estimated treatment‐covariate interaction and its confidence interval are stored. This is repeated *m* times (ideally thousands). Based on a traditional frequentist paradigm, power can then be estimated by calculating the proportion of times the summary estimate was statistically significant (eg, as defined by the associated 95% confidence interval excluding the null value). This process might also be repeated assuming different values for the size of the assumed treatment‐covariate interaction, and different values for the assumed magnitude of heterogeneity in the interaction across studies (starting from zero).

For an IPD meta‐analysis of *S* randomized trials with continuous outcomes, closed‐form solutions are obtainable for the power to evaluate a treatment‐covariate interaction.^10^ Simmonds and Higgins provide the following analytic solution for the maximum likelihood estimate of a treatment‐covariate interaction for a continuous outcome in a single randomized trial with two parallel groups:^10^

$${\hat{\gamma }}_{W\;i}=\frac{2}{\sum_{j=1}^{n_{i}}z_{\mathrm{ij}}^{\prime 2}}\left(\sum_{j\in T_{i}}y_{\mathrm{ij}}z_{\mathrm{ij}}^{\prime }-\sum_{j\in C_{i}}y_{\mathrm{ij}}z_{\mathrm{ij}}^{\prime }\right).$$

Here, *T*_*i*_ denotes the treatment group and *C*_*i*_ the control group in study *i*, and $z_{\mathrm{ij}}^{\prime }$ denotes that each *z*_*ij*_ is centered about the study‐specific mean *z*_*ij*_ value (ie, $z_{\mathrm{ij}}^{\prime }=z_{\mathrm{ij}}-{\bar{z}}_{i}$). Simmonds and Higgins use this to derive subsequent power calculations,^10^ assuming common residual variances across trials. If we extend their work by allowing for different residual variances in each trial ($\sigma_{i}^{2}$), the variance (var) of the interaction estimate in a particular study *i* is:

(15) $$\begin{array}{cc} \mathrm{var}\left({\hat{\gamma }}_{W\;i}\right) & =\mathrm{var}\left(\frac{2}{\sum_{j=1}^{n_{i}}z_{\mathrm{ij}}^{\prime 2}}\left(\sum_{j\in T_{i}}y_{\mathrm{ij}}z_{\mathrm{ij}}^{\prime }-\sum_{j\in C_{i}}y_{\mathrm{ij}}z_{\mathrm{ij}}^{\prime }\right)\right)\\ & \;=\frac{4}{{\left(\sum_{j=1}^{n_{i}}z_{\mathrm{ij}}^{\prime 2}\right)}^{2}}\left(\sum_{j\in T_{i}}z_{\mathrm{ij}}^{\prime 2}\;\mathrm{var}\left(y_{\mathrm{ij}}\right)+\sum_{j\in C_{i}}z_{\mathrm{ij}}^{\prime 2}\;\mathrm{var}\left(y_{\mathrm{ij}}\right)\right)\\ & \;=\frac{4\sigma_{i}^{2}}{{\left(\sum_{j=1}^{n_{i}}z_{\mathrm{ij}}^{\prime 2}\right)}^{2}}\left(\sum_{j\in T_{i}}z_{\mathrm{ij}}^{\prime 2}+\sum_{j\in C_{i}}z_{\mathrm{ij}}^{\prime 2}\right). \end{array}$$

Let us assume an equal number of participants in the treatment and control groups, and that the variance of the covariate ($\sigma_{z_{i}}^{2}$) is the same in each treatment group (ie, ${\sigma_{T_{i}z_{i}}^{2}=\sigma_{C_{i}z_{i}}^{2}=\sigma }_{z_{i}}^{2}$). Then $\sum_{j=1}^{n_{i}}z_{\mathrm{ij}}^{\prime 2}=n_{i}\sigma_{z_{i}}^{2}$, and Equation (15) simplifies to:

(16) $$\mathrm{var}\left({\hat{\gamma }}_{W\;i}\right)=\frac{4\sigma_{i}^{2}}{{\left(\sum_{j=1}^{n_{i}}z_{\mathrm{ij}}^{\prime 2}\right)}^{2}}\left(\sum_{j=1}^{n_{i}}z_{\mathrm{ij}}^{\prime 2}\right)=\frac{4\sigma_{i}^{2}}{\sum_{j=1}^{n_{i}}z_{\mathrm{ij}}^{\prime 2}}=\frac{4\sigma_{i}^{2}}{n_{i}\sigma_{z_{i}}^{2}}.$$

Using the solution for $\mathrm{var}\left({\hat{\gamma }}_{W\;i}\right)$ from either Equation 15, 16, we can derive a closed‐form solution for the variance of the summary interaction estimate from the second stage of a two‐stage IPD meta‐analysis. Assuming common‐effect model (4), $\mathrm{var}\left({\hat{\gamma }}_{W}\right)$ is simply the sum of the inverse of the variances from each study,

(17) $$\mathrm{var}\left({\hat{\gamma }}_{W}\right)={\left(\sum_{i=1}^{S}\frac{1}{\mathrm{var}\left({\hat{\gamma }}_{W\;i}\right)}\right)}^{-1},$$

and the subsequent power to estimate a treatment‐covariate interaction of size *γ*_*W*_ using the IPD meta‐analysis is approximately,

(18) $$\text{Power}=\text{Prob}\left(\frac{{\hat{\gamma }}_{W}}{\sqrt{\mathrm{var}\left({\hat{\gamma }}_{W}\right)}}>1.96\right)+\text{Prob}\left(\frac{{\hat{\gamma }}_{W}}{\sqrt{\mathrm{var}\left({\hat{\gamma }}_{W}\right)}}<-1.96\right),$$

For example, assuming a common interaction and that trials have equally sized groups, we can use Equations (16) and (17) to give,

(19) $$\begin{array}{cc} \text{Power} & =\text{Prob}\left(\frac{{\hat{\gamma }}_{W}}{\sqrt{\mathrm{var}\left({\hat{\gamma }}_{W}\right)}}>1.96\right)+\text{Prob}\left(\frac{{\hat{\gamma }}_{W}}{\sqrt{\mathrm{var}\left({\hat{\gamma }}_{W}\right)}}<-1.96\right)\\ & =\Phi \left(-1.96+\gamma_{W}\sqrt{\sum_{i=1}^{S}\frac{\;n_{i}\sigma_{z_{i}}^{2}}{4\sigma_{i}^{2}}}\right)\\ & \;+\Phi \left(-1.96-\gamma_{W}\sqrt{\sum_{i=1}^{S}\frac{\;n_{i}\sigma_{z_{i}}^{2}}{4\sigma_{i}^{2}}}\right). \end{array}$$

where Φ(*x*) is the probability of sampling a value < *x* from the standard normal distribution. Allowing for different sized groups in a trial (ie, using Equation (15) rather than (16)), the power calculation becomes,

(20) $$\text{Power}=\Phi \left(-1.96+\gamma_{W}\sqrt{\sum_{i=1}^{S}\left(\frac{{\left(\sum_{j=1}^{n_{i}}z_{\mathrm{ij}}^{\prime 2}\right)}^{2}}{4\sigma_{i}^{2}\left(\sum_{j\in T_{i}}z_{\mathrm{ij}}^{\prime 2}+\sum_{j\in C_{i}}z_{\mathrm{ij}}^{\prime 2}\right)}\right)}\right)+\Phi \left(-1.96-\gamma_{W}\sqrt{\sum_{i=1}^{S}\left(\frac{{\left(\sum_{j=1}^{n_{i}}z_{\mathrm{ij}}^{\prime 2}\right)}^{2}}{4\sigma_{i}^{2}\left(\sum_{j\in T_{i}}z_{\mathrm{ij}}^{\prime 2}+\sum_{j\in C_{i}}z_{\mathrm{ij}}^{\prime 2}\right)}\right)}\right),$$

and as before we could replace $\sum_{j=1}^{n_{i}}z_{\mathrm{ij}}^{\prime 2}$ with $n_{i}\sigma_{z_{i}}^{2}$, and similarly $\sum_{j\in T_{i}}z_{\mathrm{ij}}^{\prime 2}$ and $\sum_{j\in C_{i}}z_{\mathrm{ij}}^{\prime 2}$ could be replaced with${\;n_{T_{i}}\sigma }_{T_{i}z_{i}}^{2}$ and ${\;n_{C_{i}}\sigma }_{C_{i}z_{i}}^{2}$, respectively.

Equations (19) and (20) require the user to specify a minimally important value for *γ*_*W*_, and also the values of *n*_*i*_, $\sigma_{i}^{2}$, and $\sigma_{z_{i}}^{2}$ based on published study information (eg, from the baseline characteristics table for a trial) or provided directly by study authors. The residual variance ($\sigma_{i}^{2})$ might be unavailable, and so approximated by the variance of outcome values.

Extension can be made to allow for potential heterogeneity in the treatment‐covariate interaction, but the amount of heterogeneity is difficult to know in advance. Thus we suggest to focus on the power in an ideal situation where there is no between‐study heterogeneity in the size of the interaction.

#### 5.2.1 Application to the pregnancy example

In the IPD meta‐analysis conducted by the i‐WIP collaboration introduced in Section 2.2, a primary objective was to examine a potential interaction between baseline BMI and intervention effect on gestational weight gain. The prior hypothesis was that those with high baseline BMI may benefit most from weight management interventions. No formal power calculation was performed in advance of IPD collection but ultimately IPD from 24 trials. For illustration, here we reconstruct the power calculation for this project assuming it was known that IPD could be obtained from these 24 trials. The values of *n*_*i*_, $\sigma_{i}^{2}$, and $\sigma_{z_{i}}^{2}$ were extracted from the publications of the 24 trials, and are shown by Ensor et al^41^; occasionally $\sigma_{i}^{2}$ and $\sigma_{z_{i}}^{2}$ were missing and for these we used a weighted average of values from other studies. These values were input into Equation (16) to derive $\mathrm{var}\left({\hat{\gamma }}_{W\;i}\right)$ for each trial, which then were used within Equation (17) to give $\mathrm{var}\left({\hat{\gamma }}_{W}\right)=0.0014$.

A minimally important interaction size of −0.1 was suggested by clinical experts, such that the reduction in weight is at least 1 kg larger for a 10‐unit increase in BMI. Thus inputting *γ*_*W*_ =  − 0.1 and $\mathrm{var}\left({\hat{\gamma }}_{W}\right)=0.0014$ into Equation (19), we obtain:

$$\begin{array}{cc} \text{power}\; & =\Phi \left(-1.96-0.1\sqrt{\frac{1}{0.0014}}\right)+\Phi \left(-1.96+0.1\sqrt{\frac{1}{0.0014}}\right)\\ & =\Phi \left(-4.917\right)+\Phi \left(0.997\right)\\ & =0+0.84. \end{array}$$

Thus the estimated power is 84%. Ensor et al estimate a comparable 83% power using a simulation‐based power calculation,^41^ but our closed‐form solution is obtained in a much quicker time‐frame. Had this power been known at the time of the i‐WIP grant application, it would have given further merit to undertaking and funding the IPD meta‐analysis project. Although, even if power was deemed low, there are often many other potential benefits of undertaking an IPD meta‐analysis project (eg, obtaining additional follow‐up, standardizing inclusion criteria, and so on).

## 6 DISCUSSION

There are multiple reasons for pursuing the collection and synthesis of IPD, 70, 71 but personalized medicine is driving many IPD meta‐analyses to search for treatment‐covariate interactions. To guide such IPD meta‐analysis projects, this article has outlined key statistical methods for their conduct and planning, whilst drawing attention to issues and pitfalls. We focused on interactions in randomized trials, but key points also apply to modeling interactions in IPD meta‐analyses of study types, such as those evaluating the accuracy of diagnostic tests or the association of prognostic factors to subsequent outcomes.^72^

The actual identification, validation, and successful implementation of treatment‐covariate interaction is rare.^2^ A sensible starting point—for funders, researchers, health professionals, and patients—is that the relative treatment effect is similar for all individuals, unless there is strong justification otherwise (eg, from previous findings, and especially biological rationale).^11^, ^73^ We highlighted many issues and pitfalls that if ignored may produce misleading conclusions, and we suspect many will affect the 102 IPD meta‐analyses identified by Schuit et al as providing a significant interaction estimate.^74^

Our work focuses on statistical models for estimating an interaction. However if we are willing to make clinical decisions (and even withhold treatment) based on an individual's predicted treatment effect, then there should be a firm understanding of the causal mechanism and pathway, and the cost‐effectiveness of the approach. Sun et al suggest criteria to assess the credibility of a treatment‐covariate interaction (which they call a subgroup effect),^12^ including an item about the need for evidence to be based on within‐study rather than across‐study information. A key role is to disentangle different sources of variation,^75^ and Senn recommends we need more *N*‐of‐1 trials,^76^ to repeatedly test multiple treatments in the same person, including the same treatment multiple times. For further consideration of whether interactions are likely to be genuine, we refer to two excellent tutorials which discuss intricate issues such as dose‐response relationships, measurement error, adjusting for confounders, dealing with multiple covariates, and multiplicative vs additive scales.^72^, ^77^ Scale of the analysis is an important issue. For example, a treatment‐covariate interaction may occur on the odds ratio scale but not the risk ratio scale, when the covariate is also a prognostic factor, as superbly illustrated by Shrier and Pang.^78^ Conversely the risk ratio scale may also be problematic in some particular situations as, unlike the odds ratio, its value may be bounded.^45^ Nonlinear trends may also exist on one scale but not another. Therefore consideration of multiple scales may be important, as illustrated in the example of Section 4.3. Allowing for nonproportional hazards may also be important for similar reasons.

More research is needed to help address the issue of multiple testing and exploratory research when investigating multiple interactions in IPD meta‐analysis settings, extending recommendations for a single trial.^79^ Mistry et al propose a tree‐based recursive partitioning algorithm (called “IPD‐SIDES”) to identify subgroup effects in an IPD meta‐analysis when there are many covariates of interest (ie, in an exploratory analysis).^80^ A limitation of their proposal is that it requires the use of cut‐points to dichotomize continuous covariates, rather than leaving them as continuous, and—as far as we can tell—amalgamated within‐study and across‐study information. Extension of their work to address these issues would be welcome.

Treatment‐covariate interactions correspond to changes across individuals in the treatment effect as measured on the relative scale (eg, risk ratio, odds ratio, hazard ratio). However, another approach to personalizing or stratifying the use of treatments is to consider their impact on *absolute* risks. Those people with the highest absolute risk will derive the largest absolute benefit from a treatment (eg, greatest reduction in probability of the outcome) when the treatment effect expressed as a risk ratio is the same for all patients. Therefore, even in the absence of treatment‐covariate interactions, treatment decisions might be tailored conditional on absolute outcome risk.^1^, ^2^ Lastly, we note that those considering IPD meta‐analysis projects should also consider other practicalities aside from the analysis, such as obtaining, cleaning and harmonizing the IPD.^17^, ^18^

## Data Availability

The IPD used to provide examples is not routinely available for sharing
